# *Achyranthes japonica* extracts supplementation to growing pigs positively influences growth performance, nutrient digestibility, fecal microbial shedding, and fecal gas emission

**DOI:** 10.5713/ajas.20.0012

**Published:** 2020-04-13

**Authors:** Xiao Liu, Sang In Lee, In Ho Kim

**Affiliations:** 1 Department of Animal Resource and Science, Dankook University Cheonan 31116, Korea; 2 Institute of Animal Nutrition, Northeast Agricultural University, Harbin 150030, China; 3 Department of Animal Biotechnology, Kyungpook National University, Sangju 37224, Korea

**Keywords:** *Achyranthes japonica* Extracts, Growth Performance, Nutrient Digestibility, Fecal Microbial Shedding, Fecal Gas Emissions, Growing Pigs

## Abstract

**Objective:**

An experiment was conducted to investigate the effects of *Achyranthes japonica* extracts (AJE) on the growth performance, nutrient digestibility, fecal microbial shedding, and fecal gas emission of growing pigs.

**Methods:**

A total of 180 ([Landrace×Yorkshire]×Duroc) growing pigs with initial body weight (BW) of 23.94±1.54 kg were used in this study to investigate the effects of AJE as a feed additive. Dietary treatments included: i) CON (basal diet), ii) TRT1 (basal diet+0.05% AJE), and iii) TRT2 (basal diet+0.10% AJE).

**Results:**

As a result of the dietary supplementation of 0% to 0.10% AJE, a linear increase of BW (p<0.05) on d 21 and 42, a linear increase of average daily gain (ADG) (p<0.05) during d 21 to 42, a trend in linear increase of ADG (p<0.10) during d 0 to 21 and d 0 to 42, a linear increase of gain to feed ratio (G:F) (p<0.05) during d 0 to 42, and a tendency in the linear increase of G:F during d 21 to 42 were observed in this study. Additionally, dietary supplementation of 0% to 0.10% AJE had a linear increase (p<0.05) on the apparent total tract digestibility of dry matter (DM) and energy, a linear increase (p<0.05) on lactic acid bacteria counts, a tendency in reducing (linear effect, p<0.10) coliform bacteria counts, and a linear decrease (p<0.05) in excreta H_2_S emission content in growing pigs.

**Conclusion:**

In conclusion, the results suggested that AJE had the potential to enhance growth performance, DM and energy digestibility, and fecal lactic acid bacteria counts, and decrease the fecal coliform bacteria counts and excreta H_2_S emission in growing pigs.

## INTRODUCTION

With the ban on the use of antibiotics as growth promoters for animals, the development of viable antibiotic alternatives in feed is becoming an urgent need, and active research is under way worldwide [1]. Natural growth promoters, including medicinal plants and herbs, have attracted and gained widespread attention. These include a wide variety of phytochemicals such as phenolics, flavonoids, and tannins, which can play an important role in modulating nutritional metabolism [2], stimulating immune responses [3], and increasing the intestinal health of pigs [4]. In addition, various studies indicated that herbal extracts positively enhanced growth performance, improved feed efficiency, modified immune-related blood characteristics [5], and decreased the fecal noxious gas content in pigs [6].

*Achyranthes japonica* is a perennial member of the *Achyranthes* genus in the Amaranthaceae family. As a traditional medicine in South Korea to treat hypertension, rheumatism, osteoarthritis, and as an analgesic and diuretic, it is widely distributed in South Korea, China, Japan, and other East Asian countries. *Achyranthes japonica* contains multiple active components, such as saponin, triterpenoids, phytoecdysteroid, 20-hydroxyecdysone, and inokosterone [7,8]. Previous studies observed various positive physiological effects of *Achyranthes japonica*, including anti-allergic, hepato-protective, anti-inflammatory, antioxidant, arthritis alleviation, and anti-cancer properties [9–12]. Considering the above effects, *Achyranthes japonica* extracts (AJE) might be similar with other herbal extracts, which may regulate the balance of intestinal microbial flora, thus promoting the absorption of nutrients, improving growth performance, and reducing noxious gas emissions in pigs.

Additionally, the growing phase is a very important transition between the weanling and fattening phases of pigs. However, the evaluation of AJE supplementation as a growth promoter in the growing pig diet is limited. Therefore, this study evaluated the effects of AJE on the growth performance, nutrient digestibility, fecal microbial shedding, and fecal gas emission of growing pigs.

## MATERIALS AND METHODS

### Animal care

In the experiment, the experimental protocols for management and care of animals were approved by the Animal Care and Use Committee of Dankook University, South Korea (DK-4-1805).

### Product preparation

The AJE used in this study was kindly provided by a commercial company (Synergen Inc., Bucheon, Korea). The *Achyranthes japonica* was cultivated in South Korea. After wash, the roots of *Achyranthes japonica* were powdered with a mill (IKAM20; IKA, Staufen, Germany) and dried. The initial extracts were extracted with distilled water. The extract solution was filtered at −4°C by a high-velocity centrifugal machine. The useful parts were collected by column and eluted with ethanol. The finial extracts were completely dried in a freeze-drier.

### Experimental design, animals, and diets

In total, 180 ([Landrace×Yorkshire]×Duroc) growing pigs (female and castrated male) with an initial body weight (BW) of 23.94±1.54 kg were used in a 6-wk feeding trial. Based on the BW and sex, pigs were assigned to 1 of 3 dietary treatments in a randomized complete block design. The dietary treatments included: i) CON (basal diet), ii) TRT1 (CON+ 0.05% AJE), and iii) TRT2 (CON+0.10% AJE). Each treatment had 12 replicate pens with 5 pigs (3 barrows and 2 gilts) per pen. All treatment diets were formulated to meet or exceed the requirement recommended of NRC (2012) [13] for growing pigs (Table 1). The 0.05% and 0.10% AJE were mixed properly using a mixer (DDK-801F, Daedong Tech, Anyang, Korea). To allow the pig *ad libitum* access to feed and water throughout the experiment, each pen was provided with a one-side self-feeder and a nipple drinker.

### Sampling and measurements

The BW of pigs was measured on a pen basis on d 21 and d 42, and the feed consumption was recorded throughout the whole experiment. The average daily gain (ADG), average daily feed intake (ADFI), and gain to feed ratio (G:F) were calculated. During wk 6, 0.2% of chromium oxide (Cr_2_O_3_) as an inert indicator was mixed in the feed to calculate the apparent total tract digestibility (ATTD) of dry matter (DM), nitrogen (N), and gross energy. At the end of the animal trial, fresh feces were taken from all the pigs by via rectal massage, and samples were pooled and mixed on a pen basis. For nutrient digestibility, the feed and fecal samples were stored at −20°C until further analysis. For fecal microbial analyzing, fecal samples were placed on ice and immediately transported to the laboratory for the microbial analysis. For fecal gas emission, fresh urine was collected with a bucket via a funnel below the pens. Feces and urine were mixed well (1:1 on wet weight basis) within a pen and stored in a 2.6 L plastic box in duplicates for the fermentation and further analysis. Each box had a small hole in the middle of one sidewall that was sealed with adhesive tape.

### Chemical analysis

For the nutrient digestibility analysis, fecal samples were dried at 70°C for 72 h and finely ground to pass through a 1-mm screen first. The feed and fecal samples were analyzed for DM (method 930.15) and N (method 990.03) according to the Association of Official Analytical Chemists (AOAC, 2000) [14]. Gross energy was determined by measuring the heat of combustion in the samples using a Parr 6100 oxygen bomb calorimeter (Parr instrument Co., Moline, IL, USA). Chromium was analyzed by using UV absorption spectrophotometry (Shimadzu, UV-1201, Kyoto, Japan). The ATTD was then calculated from the following formula: digestibility (%) = [1–([Nf×Cd]/[Nd×Cf])]×100, where, Nf is nutrient concentration in feces (% DM), Nd is nutrient concentration in diet (% DM), Cd is chromium concentration in diet (% DM), and Cf is chromium concentration in feces (% DM).

One gram of fecal sample for fecal microbial was diluted with 9 mL of 1% peptone broth (Becton, Dickinson and Co., Franklin Lakes, NJ, USA) and homogenized. Viable counts of bacteria in the fecal samples were then conducted by plating serial 10-fold dilutions (in 1% peptone solution) onto Mac Conkey agar plates (Difco Laboratories, Detroit, MI, USA) for coliform bacteria isolation and onto *Lactobacillus* medium III agar plates (Medium 638, DSMZ, Braunschweig, Germany) for lactic acid bacteria isolation. The *Lactobacillus* medium III agar plates were then incubated for 48 h at 39°C under anaerobic conditions. The selective medium for coliform bacteria was MacConkey agar. The MacConkey agar plates were incubated for 24 h at 37°C. After removal from the incubator, the coliform bacteria and lactic acid bacteria colonies were counted immediately.

Before chemical analysis for fecal noxious gas emission of ammonia (NH_3_), hydrogen sulfide (H_2_S), and total mercaptans, the samples were allowed to ferment for 7 days at 25°C. After fermentation, the concentration of NH_3_, H_2_S, and total mercaptans were determined by using a GV-100 gas sampling pump (Gastec Corp., Kanagawa, Japan) with different detection tubes (No. 3 L, No. 4LT, and No. 70 L; Gastec, Japan). One hundred mL of the headspace air was sampled about 2 cm above the slurry. Two samples from one pen were measured and calculate the average values.

### Statistical analysis

All data were subjected to the MIXED procedures of SAS (SAS Inst. Inc., Cary, NC, USA), with the following statistical model: *Y_ijk_* = *μ*+*t_i_*+*r_k_*+*e_ijk_*, where *Y_ijk_* was an observation on the dependent variable *ij*, *μ* was the overall population mean, *t_i_* was the fixed effect of AJE treatments, *r_k_* was the pen as a random effect, and *e_ijk_* was the random error associated with the observation *ijk*. Orthogonal polynomials were used to assess the linear and quadratic effects of increasing dietary concentrations of supplemental AJE. A probability value of p<0.05 was considered statistically significant and trends were noted under conditions of 0.05<p<0.10.

## RESULTS

### Growth performance

Throughout the experiment, ADFI was not affected by treatments. On d 21 and 42, pigs fed AJE diets linearly increased BW (p<0.05). Dietary AJE supplementation linearly increased ADG during d 21 to 42 (p<0.05) and showed a trend in linear increase in ADG during d 0 to 21 and d 0 to 42 (p<0.10) as the dietary AJE supplementation increased from 0% to 0.10%. Increasing dietary supplementation of AJE led to a linear increase in the G:F during d 0 to 42 (p<0.05), and a tendency in the linear increase of G:F during d 21 to 42 (p<0.10; Table 2).

### Nutrient digestibility

The ATTD of DM and energy increased linearly in pigs fed diets supplemented with 0% to 0.10% AJE (p<0.05). No treatment effects were observed on the ATTD of nitrogen (Table 3).

### Fecal microbial shedding

Fecal lactic acid bacteria counts showed a linear increase (p< 0.05), and fecal coliform bacteria counts tended to reduce linearly (p<0.10) with the dietary AJE supplementation increased from 0% to 0.10% (Table 4).

### Fecal gas emission

There was a linear decrease (p<0.05) in fecal H_2_S emission with the dietary AJE supplementation increased from 0% to 0.10%, no differences were observed in fecal NH_3_ and total mercaptans emission (Table 5).

## DISCUSSION

Due to the lack of studies on the efficacy of AJE in pigs, this study attempted to evaluate the effects of AJE as a natural growth promoter for growing pigs. In this study, BW, ADG, and G:F of growing pigs fed with AJE showed positive effects. Consistent with the current study, some herbs and plant extracts had been evaluated as growth promoters or immune enhancers and as dietary supplements, which also have indicated a potential to improve growth performance in pig production [6,15–17]. The exact mechanisms of the relationship between AJE and improvement in growing pig growth performance are still unclear. However, it is well known that *Achyranthes japonica* has many physiological and biochemical functions. It is rich in active ingredients that have antioxidant and bioactive properties, such as saponin, triterpenoids, phytoecdysteroid, 20-hydroxyecdysone, and inokosterone [7,18]. Meng and Li [19] reported that *Achyranthes japonica Nakai* extracts have many biological functions, such as anti-inflammatory, hepato-protective, antioxidant, and anti-cancer activities. Previous studies also have shown that herbal feed additives, such as a wide range of spices, herbs, and extracts, have been demonstrated to improve digestive tract function by increasing the activity of digestive enzymes of gastric mucosa and the nutrient utilization of animals [20,21]. In the current study, supplementation of AJE to the diets may have helped to stimulate mucus secretion in the intestinal tract, prevented the adhesion of pathogens, and contributed to the stabilization of favorable microbiota. Thereby, AJE may facilitate better digestion and absorption of nutrients by pigs, consequently improving growth performance. Meanwhile, the ATTD of DM and energy increased linearly in growing pigs fed AJE diets in the current study. The results indicated that higher concentrations of AJE could obtain higher digestibility. Similarly, Yan et al [6] reported that the supplementation of herb (*Houttuynia cordata*) extract increased growth performance and the ATTD of DM in pigs. Although this study did not test the intestinal morphology, previous studies have shown that villus height was increased and crypt depth was decreased in the intestine in response to *Achyranthes bidentata* extracts [22,23]. Increased villus height and decreased crypt depth are usually associated with effective nutrient absorption and better performance. These might be the reasons why the DM and energy digestibility are improved. However, the influence of the mechanism of action needs to be further researched. In addition, during the whole experiment, the ATTD of energy and DM increased by the supplementation with AJE, which may be the reason for improving the growth performance of growing pigs.

An overabundance of coliform bacteria in the gastrointestinal system causes diarrhea, which leads to a decline in the growth performance in domesticated animals [24]. Moreover, lactic acid bacteria could adjust the gut microbiota because it produces broad-spectrum bacteriocins and facilitates the elimination of various enteropathogens [25]. Therefore, fecal coliform bacteria and lactic acid bacteria counts, are indicators of the microflora balance of the gastrointestinal tract, which plays an important role in gastrointestinal health. In the current study, dietary supplementation with AJE increased the counts of lactic acid bacteria and decreased the counts of coliform bacteria. Similar results were observed by Chen et al [26], who reported that various doses of *Achyranthes bidentata* extract supplementation significantly reduced the diarrhea frequency of weaned piglets, suggesting an inhibition effect on gut pathogens. As there have not been enough studies on the use of AJE in pigs, the present results have to be compared with studies on herbs or other plant extracts. Yan et al [27] showed that weaning pigs fed herbal extract mixtures as supplemental diets decreased the fecal *Escherichia coli* concentration. Additionally, Xie et al [28] indicated that dietary supplementation with *Achyranthes bidentata* extract in pigs promotes the growth of *Lactobacillus*. *Achyranthes* plant extracts could exhibit antioxidative properties, antimicrobial activity, and immunostimulating effects [23]. Furthermore, herbs could inhibit the growth of pathogenic microorganisms in the gastrointestinal tract, thus improving animals’ resistance ability to different stress situations [29]. These could be the possible explanations of the decreasing coliform bacteria counts and increasing lactic acid bacteria counts in this study. The decreased coliform bacteria counts and increased lactic acid bacteria counts may also serve as further evidence to explain the improved growth performance and nutrient digestibility.

In the swine industry, a serious environmental problem is the production of harmful gases, and NH_3_, H_2_S, and total mercaptan are the main air pollutants in the pig production process. Previously, Ferket et al [30] indicated that the release of excreta noxious gases from animal feces is associated with intestinal microflora, particularly harmful intestinal bacterial populations, such as *Escherichia coli*. Yin et al [31] indicated that the inclusion of herbal (red ginseng) extract manipulated the microflora in the gastrointestinal tract of pigs, thereby reducing the fecal noxious gas content in pigs. In contrast, Yan et al [32] reported that the reduced fecal noxious gas content might be due to increased nutrient digestibility in pigs. The increased nutrients digestibility could lead to less substrate for the microbial fermentation, which consequently decreases noxious gas emission [33]. Consistent with previous studies, dietary supplementation of AJE linearly decreased the fecal H_2_S emission and coliform bacteria counts and increased the DM and energy digestibility in the current study. There is currently a lack of data regarding the usefulness of AJE in pigs. Similar to this study, Cho et al [34] demonstrated that fecal H_2_S concentration of pigs fed diets supplemented with essential oils was lower than control diets. In addition, Yan et al [6] indicated that herbal extract mixtures supplementation decreases the fecal H_2_S concentration compared with the control diets. Therefore, it is suggested that the reason for the decreasing fecal H_2_S gas content may be the result of the increased nutrient digestibility and the decreased the coliform bacteria counts in growing pigs. Further research is needed to determine the exact mechanisms of the association between AJE and fecal gas emission in pigs.

In conclusion, with the AJE supplementation there were increases in growth performance, DM and energy digestibility, fecal lactic acid bacteria, and decreases in coliform bacteria counts and fecal H_2_S emissions. It is suggested that the use of AJE as a feed additive may exert beneficial effects in pigs. More research is needed to determine the mechanisms underlying the effect of AJE.

## Figures and Tables

**Table 1:** Composition of experimental diets (as-fed basis)

Items	
Ingredients (%)	
Corn	60.37
Soybean meal	16.07
Wheat	6.00
Rapeseed meal	2.50
Distillers dried grains with solubles	6.50
Tallow	3.00
Molasses	3.00
Dicalcium phosphate	1.08
Limestone	0.65
Salt	0.30
L-Lysine-HCl (78.8%)	0.19
Vitamin premix1)	0.20
Mineral premix2)	0.10
Choline	0.04
Calculated composition (%)	
Crude protein	15.50
Crude fat	5.78
Crude fiber	3.43
Crude ash	4.59
Lysine	0.91
Standardized ileal digestible lysine	0.59
Calcium	0.65
Total phosphorus	0.55
Available phosphorus	0.34
Digestible energy (kcal/kg)	3,428

1) Provided per kg of complete diet: 11,025 IU vitamin A; 1,103 IU vitamin D_3_; 44 IU vitamin E; 4.4 mg vitamin K; 8.3 mg riboflavin; 50 mg niacin; 4 mg thiamine; 29 mg D-pantothenic acid; 166 mg choline; 33 μg vitamin B_12_.

2) Provided per kg of complete diet: 12 mg Cu (as CuSO_4_·5H_2_O); 85 mg Zn (as ZnSO_4_); 8 mg Mn (as MnO_2_); 0.28 mg I (as KI); 0.15 mg Se (as Na_2_SeO_3_·5H_2_O).

**Table 2:** Effect of dietary supplementation *Achyranthes japonica* extracts on growth performance in growing pigs

Items	CON1)	TRT11)	TRT21)	SEM	p-value	

					Linear	Quadratic
Body weight (kg)						
Initial	23.94	23.94	23.94	0.03	0.997	0.996
Days 21	37.52	37.81	38.03	0.12	0.006	0.833
Days 42	52.48	53.12	53.70	0.30	0.006	0.927
Days 0–21						
ADG (g)	646	660	671	10	0.085	0.892
ADFI (g)	1,320	1,334	1,340	26	0.578	0.904
G:F	0.490	0.495	0.501	0.005	0.140	0.918
Days 21–42						
ADG (g)	713	729	746	11	0.040	0.988
ADFI (g)	1,635	1,650	1,668	27	0.404	0.956
G:F	0.436	0.443	0.448	0.004	0.058	0.880
Days 0–42						
ADG (g)	679	695	709	10	0.052	0.952
ADFI (g)	1,478	1,492	1,504	23	0.426	0.968
G:F	0.460	0.466	0.,471	0.003	0.019	0.998

SEM, standard error of the mean; ADG, average daily gain; ADFI, average daily feed intake; G:F, gain:feed; AJE, *Achyranthes japonica* extracts.

1) CON, basal diet; TRT1, CON+0.05% AJE; TRT2, CON+0.10% AJE.

**Table 3:** Effect of dietary supplementation *Achyranthes japonica* extracts on nutrient digestibility in growing pigs

Items (%)	CON1)	TRT11)	TRT21)	SEM	p-value	

					Linear	Quadratic
Dry matter	75.90	76.57	77.92	0.84	0.047	0.689
Nitrogen	75.59	75.89	76.20	0.79	0.592	0.994
Energy	63.64	64.75	65.18	0.52	0.047	0.594

SEM, standard error of the mean; AJE, *Achyranthes japonica* extracts.

1) CON, basal diet; TRT1, CON+0.05% AJE; TRT2, CON+0.10% AJE.

**Table 4:** Effect of dietary supplementation *Achyranthes japonica* extracts on fecal microbial shedding in growing pigs

Items	CON1)	TRT11)	TRT21)	SEM	p-value	

					Linear	Quadratic
Lactic acid bacteria (log CFU/g)	7.52	7.60	7.65	0.03	0.026	0.723
Coliform bacteria (log CFU/g)	6.13	6.10	6.05	0.03	0.064	0.701

SEM, standard error of the mean; AJE, *Achyranthes japonica* extracts.

1) CON, basal diet; TRT1, CON+0.05% AJE; TRT2, CON+0.10% AJE.

**Table 5:** Effect of dietary supplementation *Achyranthes japonica* extracts on fecal gas emission in growing pigs

Items (ppm)	CON1)	TRT11)	TRT21)	SEM	p-value	

					Linear	Quadratic
Ammonia	5.98	5.93	5.55	0.43	0.629	0.830
Total mercaptans	3.30	3.05	2.83	0.21	0.150	0.963
Hydrogen sulfide	3.78	3.40	3.10	0.14	0.009	0.837

SEM, standard error of the mean; AJE, *Achyranthes japonica* extracts.

1) CON, basal diet; TRT1, CON+0.05% AJE; TRT2, CON+0.10% AJE.
